# Influence of Dopaminergic Medication on Conditioned Pain Modulation in Parkinson's Disease Patients

**DOI:** 10.1371/journal.pone.0135287

**Published:** 2015-08-13

**Authors:** Wiebke Grashorn, Odette Schunke, Carsten Buhmann, Katarina Forkmann, Sabrina Diedrich, Katharina Wesemann, Ulrike Bingel

**Affiliations:** 1 Department of Neurology, University Medical Center Hamburg Eppendorf, Hamburg, Germany; 2 Clinic for Neurology at the Medical Faculty, University of Duisburg-Essen, Essen, Germany; University of Würzburg, GERMANY

## Abstract

**Background:**

Pain is highly prevalent in patients with Parkinson’s disease (PD), but little is known about the underlying pathophysiological mechanisms. The susceptibility to pain is known to depend on ascending and descending pathways. Because parts of the descending pain inhibitory system involve dopaminergic pathways, dysregulations in dopaminergic transmission might contribute to altered pain processing in PD. Deficits in endogenous pain inhibition can be assessed using conditioned pain modulation (CPM) paradigms.

**Methods:**

Applying such a paradigm, we investigated i) whether CPM responses differ between PD patients and healthy controls, ii) whether they are influenced by dopaminergic medication and iii) whether there are effects of disease-specific factors. 25 patients with idiopathic PD and 30 healthy age- and gender-matched controls underwent an established CPM paradigm combining heat pain test stimuli at the forearm and the cold pressor task on the contralateral foot as the conditioning stimulus. PD patients were tested under dopaminergic medication and after at least 12 hours of medication withdrawal.

**Results:**

No significant differences between CPM responses of PD patients and healthy controls or between PD patients “on” and “off” medication were found. These findings suggest (i) that CPM is insensitive to dopaminergic modulations and (ii) that PD is not related to general deficits in descending pain inhibition beyond the known age-related decline. However, at a trend level, we found differences between PD subtypes (akinetic-rigid, tremor-dominant, mixed) with the strongest impairment of pain inhibition in the akinetic-rigid subtype.

**Conclusions:**

There were no significant differences between CPM responses of patients compared to healthy controls or between patients “on” and “off” medication. Differences between PD subtypes at a trend level point towards different pathophysiological mechanisms underlying the three PD subtypes which warrant further investigation and potentially differential therapeutic strategies in the future.

## Introduction

Pain is highly prevalent in patients with Parkinson’s disease (PD). It affects up to 83% of patients [1], often precedes motor symptoms [2, 3] and impairs patients’ quality of life [4]. Although Charcot described pain in PD already in 1878 [5] only little is known about the underlying neurophysiological mechanisms.

The susceptibility to pain is supposed to depend on the balance of activity in ascending and descending pain pathways [6–8]. The descending pain control system modulates pain by inhibiting or facilitating nociceptive processing [6, 8]. Well-established tools to study this system in humans are conditioned pain modulation (CPM) paradigms in which pain intensity ratings of test stimuli are obtained in the presence and absence of a concomitantly, remotely applied conditioning stimulus [9]. Positive CPM responses (= reduced pain intensity ratings under concurrent stimulation) are indicative of endogenous analgesia and are mediated by spino-bulbo-spinal reflexes [10, 11] which are controlled by higher cortical brain areas [12–14].

To date, pathophysiological hypotheses regarding pain in PD mainly focus on basal ganglia dysfunction [15, 16]. However, neurodegeneration in PD has been found to affect brain regions other than the basal ganglia, progressing from the olfactory bulb and inferior brain stem to midbrain and finally meso- and neo-cortical areas [17]. Given that neurodegeneration involves both brainstem and cortical areas relevant for descending pain modulation [18], aberrant descending pain inhibition might contribute to altered pain processing in PD.

To our knowledge only two studies have investigated descending pain control in PD so far. Both reported no significant differences in CPM magnitude in PD patients compared to controls [19, 20]. Moreover, no effect of dopaminergic medication on CPM response [19] has been found.

Other studies investigating the influence of dopaminergic medication on pain processing in PD yielded mixed results with a trend for anti-nociceptive properties of dopamine [18, 21–24]. Because parts of the descending pain inhibitory system involve dopaminergic pathways (i.e., rostral agranular insular cortex, dorsal horn neurons) [25, 26], dysregulations in dopaminergic transmission might contribute to altered pain processing in PD.

We used a well-established CPM paradigm [12, 27] to investigate i) whether CPM responses differ between PD patients and healthy controls and ii) whether CPM responses in PD are influenced by dopaminergic medication (“on”) or a medication withdrawal of at least 12 hours (“off”). Furthermore, the influence of expectation and PD-specific factors such as the presence of chronic pain and the PD subtype were assessed which have not been evaluated previously.

## Methods

### Participants

PD patients were recruited from our movement disorders outpatient clinic (head: Prof. Carsten Buhmann) of our department of neurology of the University Medical Center Hamburg-Eppendorf and had to fulfill the following inclusion criteria: (1) diagnosis of idiopathic PD according to the UK PD Society Brain Bank criteria, (2) Hoehn & Yahr scale < stage III [28], (3) aged between 40 and 90 years, (4) no severe cognitive impairment (Parkinson Neuropsychometric Dementia Assessment (PANDA) instrument >15 [29]), (5) no manifest depression or anxiety (Hospital Anxiety and Depression Score (HADS); subscores ≤11 [30]), (6) no acute pain or analgesic medication during the last 24 hours, (7) no history of chronic pain disorders e.g. rheumatoid arthritis (PD specific chronic pain according to Ford [31] was allowed), (8) no regular use of prescription analgesics, tranquilizers, antidepressants, pain modulating anticonvulsants (e.g. gapapentin or pregabaline), (9) no pregnancy and (10) no neuropathy (e.g. diabetic or post-chemotherapy).

Healthy controls were recruited locally and had to fulfill the same inclusion criteria except for (1) and (2).

### Ethics Statement

The study was conducted in accordance with the Declaration of Helsinki and approved by the Ethics Committee in Hamburg. All participants gave written informed consent and were free to withdraw from the study at any time.

### Experimental Protocol

**Fig 1:**
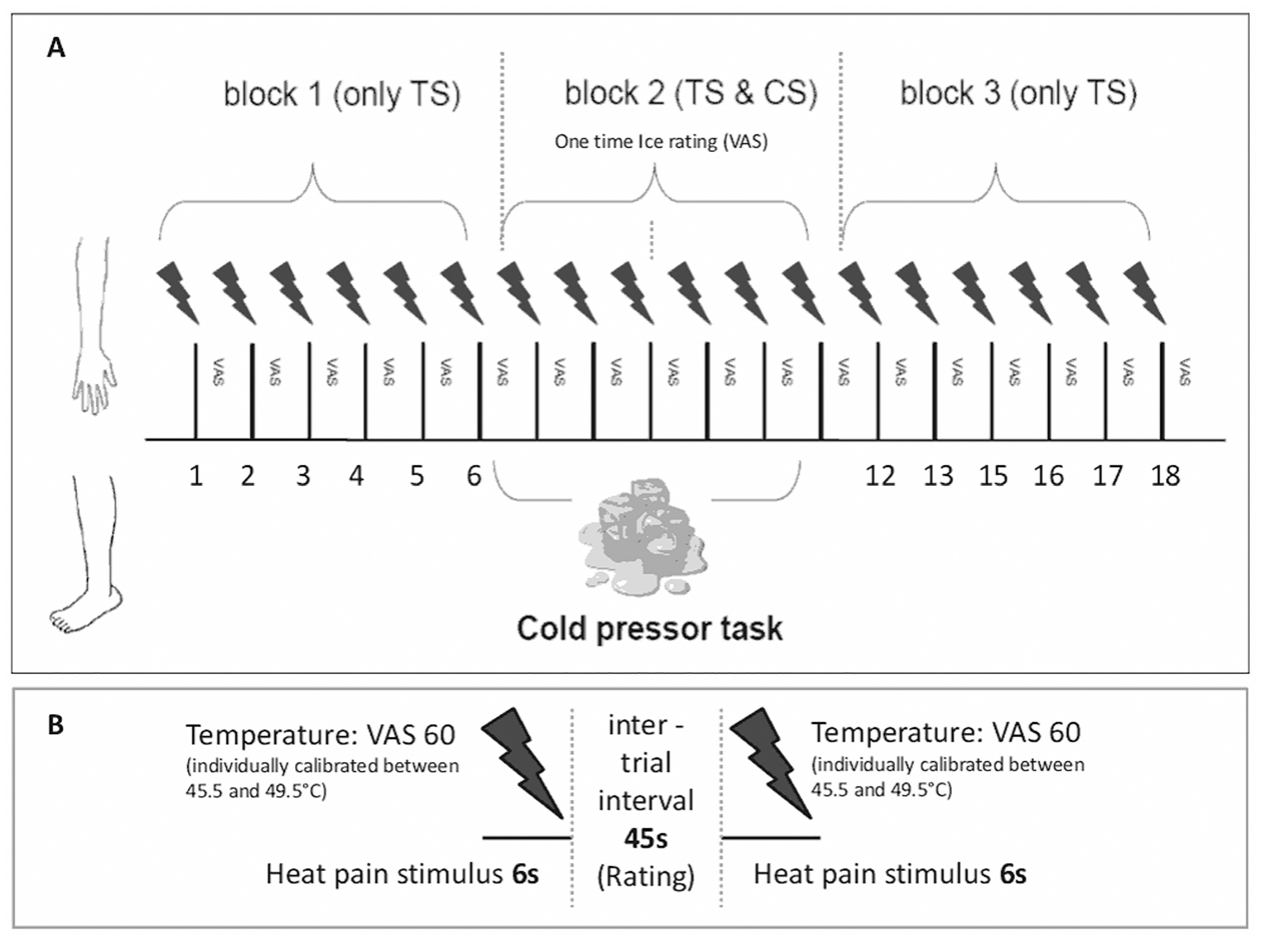
Experimental protocol. This figure shows the experimental sequences of the conditioned pain modulation (CPM) paradigm used in this study (A = whole experiment, B = temporal components of trials). During block 1 and 3, the test stimulus (TS) was applied alone whereas during block 2 the TS and a conditioning stimulus (= cold pressor task using ice water; CS) were applied concurrently. Patients had to rate the pain intensity of TS and CS on a visual analogue scale (VAS).

In this study we used a well-established CPM paradigm [12, 27] which combines painful heat stimuli as test stimuli (TS) with a cold pressor task as the conditioning stimulus (CS). In brief, the experimental procedures included an introductory session which consisted of a clinical interview, assessment of Hoehn and Yahr stage, Unified Parkinson's Disease Rating Scale and PD subtype, filling in of questionnaires and the calibration of stimulus intensities. This was followed by the a priori assessment of expectation regarding possible changes of pain intensities during the application of the cold pressor task. Finally, the actual CPM paradigm was performed, that consisted of three blocks, in which six test stimuli each were applied to the right volar forearm. Pain ratings to these stimuli were obtained before (= block I), during (= block II) and after (= block III) a cold pressor task that was applied to the contralateral leg during the second block. PD patients underwent the paradigm twice on two separate days in a counterbalanced order, once under their usual dopaminergic medication (= “on”) and again after at least 12 hours of medication withdrawal (= “off”). Controls were tested once as retest reliability of conditioned pain modulation paradigms has been shown especially for paradigms applying the cold pressor task [32]. The experimental protocol is summarized in Fig 1.

### Instructions and calibration procedure

All participants were instructed using a standardized protocol. Participants were told that the purpose of the study was to characterize possible differences in the perception of two simultaneously applied painful stimuli comparing PD patients with healthy participants of the same age. First, participants were informed about the sequence of experimental procedures. These general instructions were followed by a clinical interview (both groups) checking again all inclusion criteria e.g. asking for any chronic pain disorders e.g. rheumatoid arthritis. In the PD group an experienced clinician made a re-evaluation of PD diagnosis and then assessed the individual Hoehn and Yahr stage [28], Unified Parkinson's Disease Rating Scale (UPDRS [33]) score (total and motor score (part III)) and PD subtype. UPDRS scores were determined with and without medication to investigate the influence of medication withdrawal on motor performance. PD subtypes were classified clinically according to the German AWMF Guidelines (www.awmf.org) as tremor-dominant (n = 8), akinetic-rigid (n = 7) or mixed (n = 10) depending on the predominant motor symptom (tremor, bradykinesia/rigidity or an equal manifestation of both) that had to be predominant at symptom onset and over the course of disease.

Both groups completed the HADS [30] as depression and anxiety can modulate pain perception [34, 35] and an assessment of acute pain was performed asking the patients for any pain they might have experienced during the 24 hours prior to the experiment (in case they experienced pain during the 24 hours prior to the experiment, they were excluded from the study). The PANDA [29] tested for cognitive impairment. Subjects with scores <15 were excluded to ensure a sufficient task comprehension and compliance. If they fulfilled all inclusion criteria (see 2.1) a calibration procedure was performed to determine the individual temperatures corresponding to a pain level of 50–60 on a 0–100 visual analogue scale [VAS, endpoints 0–100]. To this end, we applied ten stimuli á six seconds each with different intensities ranging from 45.5–49.5°C in a pseudo-randomized order to the right volar forearm, every temperature was presented once. Participants were asked to rate the intensity of each stimulus on a VAS which was presented on a computer screen in front of the subjects and ranged from 0 = “no sensation” to 100 = “most intense pain imaginable”. Two vertical white lines represented the two endpoints 0 and 100 of the VAS, a third white line was set at 25 labeled as “pain threshold” to assess non-painful sensations which might occur during the cold pressor task as a result of an effective pain inhibition. Subjects indicated the pain intensity of each heat pain stimulus by moving a red bar between the two endpoints using two buttons of a computer mouse. Participants did have as much time as they needed to provide their ratings, the experiment continued only once they had made their ratings. The maximum stimulation temperature was restricted to 49.5°C in order to avoid any tissue damage. This calibration procedure ensured that all participants perceived the phasic heat pain stimuli (= test stimuli, TS) as equally painful (VAS 50–60).

The application of the thermal stimuli, the presentation of the VAS and the recording of behavioral data was performed using the software “Presentation” (www.neurobs.com).

#### Test stimulus

We used phasic heat pain stimuli as test stimuli (TS). The test stimuli were applied to the right volar forearm (~ 10 cm proximally from the wrist) of the participants using a 30x30mm Peltier-Thermode (TSAII, Medoc, Israel). Each stimulus had a duration of six seconds (baseline temperature 35°C, ramp up and down 10°C/second, destination temperature individually calibrated between 45.5 and 49.5°C, interstimulus-interval ~45 seconds). Pain ratings on the VAS were obtained immediately after each stimulus. A total of 18 test stimuli were applied. The first (= *block I*, stimulus one to six) and the last six stimuli (= *block III*, stimulus 13–18) were applied without any other concomitant procedures. During the application of test stimuli seven to twelve (= *block II*), the conditioning stimulus was applied.

#### Conditioning stimulus

A cold pressor task was used as the conditioning stimulus (CS). After completion of the first block of six heat pain stimuli (*block I*), a message on the computer screen prompted the participants to immerse their left foot into a bath with ice water (~0°C). The intensity of the conditioning stimulus was rated once in the middle of the cold pressor task (= after TS 9, block II) using a VAS presented on a computer screen with the same endpoint labels 0 = “no sensation” and 100 = “most intense pain imaginable” and a third white line set at 25 labeled as “pain threshold”. At the end of block II another message on the computer screen instructed the participants to take their foot out of the ice water. After taking their foot out of the ice water participants positioned their foot in a towel on the floor next to the tub with ice water. Prior to the experiment subjects were asked to focus their attention on the heat stimuli applied to the arm while having their foot immersed into the ice water and it was pointed out again that they could withdraw from the experiment at any time by telling the supervising experimenter. Finally heat pain stimuli 13–18 (block III) were applied without concomitant painful stimulation to the foot.

#### Assessment of individual expectation

Many cognitive and affective processes could influence CPM responses. However, expectations which are known to modulate pain have previously been suggested to affect CPM responses [36–39]. Following the calibration procedure, immediately prior to the actual experiment, patients were presented the following question on the computer screen: “How do you expect the pain applied to your arm to change while you have your foot immersed into the ice water?” Participants were asked to indicate their expectations on a computerized VAS with the verbal anchors -1 = “no sensation” (= pain at the arm would be completely abolished during the cold pressor task), 0 = “no change” (= no change of heat pain at the arm during the cold pressor task), and 1 = “maximum pain” (= pain applied to the arm would get worse during the cold pressor task). Two vertical white lines represented the two endpoints -1 (“no sensation”) and 1 (“maximum pain”) of the VAS, a third white line was set at 0 labeled as “no change”. Subjects indicated their expectation by moving a red bar between the two endpoints using two buttons of a computer mouse. Participants did have as much time as they needed to provide their ratings, the experiment continued only once they had made their ratings. As in previous studies no specific suggestions regarding the direction of possible changes were divulged [36].

#### Assessment of anxiety and depression

The Hospital Anxiety and Depression Scale (HADS) [30] is a self-report questionnaire to assess anxiety and depression with 7 items per subscale. Each item is scored from 0–3 points so that scores of 21 points for each subscale depression and anxiety can be reached, higher scores indicating higher symptom severity. Both subscales have been validated to have good sensitivity and specificity [40].

### Data analysis

Data analysis was performed using IBM SPSS 20.0. Non-parametric tests were used in case the assumptions of variance homogeneity (Levene´s test) and normal distribution (Kolmogorov-Smirnov test) were violated.

As in previous studies [41, 42] the CPM response was calculated as the difference between mean pain ratings before and after the cold pressor task and mean pain ratings during the cold pressor task (CPM response = mean pain ratings block (I+III) - blockII). A positive CPM response indicates a reduction in pain perception during the cold pressor task and therefore signifies analgesia symbolizing effective descending pain inhibition mechanisms, whereas a negative CPM response shows an increase of pain ratings in block II.

To test for significant CPM responses in healthy controls and patients (including the on and off condition) separate one sample t-tests on CPM responses were performed for the PD group in both the “on” and “off” condition and the control group.

For between-group comparisons between PD patients and controls we used two-sample t-tests and non-parametric Mann-Whitney U tests. For comparisons within the PD group (“on” vs. “off”) paired t-tests and non-parametric Wilcoxon tests were used. Kruskal-Wallis tests were used for PD subtype analyses. Correlations were calculated using Pearson’s or non-parametric Spearman’s coefficients. P-values <0.05 were considered statistically significant. Test results were corrected for multiple comparisons using Bonferroni correction.

## Results

### Clinical and neuropsychiatric assessment

10 PD patients and 2 controls did not fulfill the inclusion criteria or complete the study and were excluded: Four patients and two controls withdrew from the study during the cold pressor task (it was too painful for them), four patients did not attend the second session, one patient did not take any dopaminergic medication and one patient had H & Y score ≥ 3. The final data analysis is therefore based on 25 PD patients (*67*.*2 years+/-7*.*6 [50–86]*, *16 male*) and 30 healthy controls (*67*.*2+/-8*.*0 [51–79]*, *16 male*) matched in age (*t(53) = -0*.*019*, *p = 0*.*985*) and gender (*χ² = 0*.*638*, *p = 0*.*425*). Due to the exclusion of 10 PD patients, 16 patients were first tested “on” and 9 patients “off” medication. Patient characteristics are shown in Table 1.

**Table 1:** Characteristics of PD patients.

**Patient characteristics (n = 25)**	
Hoehn & Yahr Scale	-
	H & Y stage I: 6 patients (24%)
	H & Y stage II: 19 patients (76%)
Clinically most affected half of the body	
	right: 19 patients
	left: 6 patients
Disease duration (time since first time occurrence of symptoms prior to study)	3.7 years +/- SD 2.7 [0–12 years]
Mean age at time of symptom onset	62.1 years +/- SD 6.6 [48–73 years] (data of 1 patient is missing because he did not recognize onset)
Mean age at time of receiving PD diagnosis	63.3 years +/- SD 6.5 [48–75 years] (data of 1 patient is missing)
Number of patients with medication (n = 25)	
	DA agonist & MAO-B inhibitor: n = 9 (36%)
	DA agonist: n = 6 (24%)
	Levodopa: n = 4 (16%)
	Levodopa & MAO-B inhibitor: n = 2 (8%)
	MAO-B inhibitor: n = 2 (8%)
	>1 dopamine agonist: n = 1 (4%)
	Levodopa & DA agonist: n = 1 (4%)
UPDRS total score (data of 1 patient is missing)	
	UPDRS “on”: 29.4 +/- SD 13.0 [7–57]
	UPDRS “off”: 33.8 +/- SD 14.2 [10–61]
UPDRS motor score (data of 1 patient is missing)	
	UPDRS motor “on”: 20.7 +/- SD 8.9 [3–43]
	UPDRS motor “off”: 24.1 +/- SD 9.7 [6–43]

Patient characteristics regarding disease classification, symptom onset, medication and clinical scores such as UPDRS are shown for PD patients.

As expected, in PD patients total and motor UPDRS scores were significantly lower under medication compared to the “off” condition (p<0.001). Mean PANDA and HADS scores were comparable between PD patients and controls (Table 2).

**Table 2:** Main results for healthy subjects and Parkinson patients (PD).

Parameter (mean, standard deviation, [Min-Max])	Healthy subjects (n = 30)	Parkinson patients (n = 25)		Group comparison Healthy subjects vs. Parkinson	Inner group comparison on vs. off
		With medication (on)	Without medication (off)		
**PANDA Score**	25.1 +/- 3.3	25.9 +/- 3.0		t(53) = 0.935,	-
	[17–30]	[19–30]		p = 0.354	
**HADS subscale depression**	1.9 +/- 1.8	3.0 +/- 2.3		U = 270.50,	
	[0–6]	[0–7]		z = -1.792,	-
				p = 0.073	
**HADS subscale anxiety**	2.4 +/- 2.0	3.5 +/- 2.9		U = 308.00,	-
	[0–6]	[0–11]		z = -1.145,	
				p = 0.252	
**UPDRS Total Score** *	-	29.4 +/- 12.9	33.8 +/- 14.2	-	t(23) = -5.463,
		[7–57]	[10–61]		p < 0.001
**UPDRS Motor Score** *	-	20.7 +/- 8.9	24.1 +/- 9.7	-	t(23) = -4.605,
		[3–43]	[6–43]		p < 0.001
**Mean stimulation temperature**				on: U = 247.00,	
				z = -1.295,	
	48.1°C +/- 0.7°C	48.2°C +/- 1.0°C	48.2°C +/- 0.8°C	p = 0.195	T = 29.00,
	[47.0–49.5°C]	[46.0–49.0°C]	[46.0–49.0°C]	off: U = 253.00,	p = 0.875
				z = -0.962,	
				p = 0.336	
**Mean pain intensity Block 1 [VAS 0–100]**				on: t(53) = 1.080,	
	55.1 +/- 9.3	58.1 +/- 11.4	56.1 +/- 11.8	p = 0.285	t(24) = 0.805,
	[37.0–68.3]	[30.7–75.3]	[34.5–84.5]	off: t(53) = 0.355,	p = 0.429
				p = 0.724	
**Cold pain rating Block 2 [VAS 0–100]**				on: t(53) = -1.413,	
	71.9 +/- 21.5	63.2 +/- 24.3	59.3 +/- 23.5	p = 0.163	t(24) = 1.226,
	[23–97]	[22–99]	[14–97]	off: t(53) = -2.081,	p = 0.232
				p = 0.042†	
**Expectation Rating Day 1 [-1 to +1]**				PD total:	
				(0.002 +/- SD 0.16):	
				U = 316.50,	
	30 controls:	16 patients:	9 patients:	z = -1.039,	U = 107.50,
	-0.04 +/- 0.18	0.05 +/- 0.15	-0.08 +/- 0.13	p = 0.299	z = 2.131,
	[-0.40–0.33]	[-0.19–0.40]	[-0.29–0.14]	on: U = 162.50,	p = 0.043†
				z = -1.894,	
				p = 0.058†	
	Pain better: 12	Pain better: 2	Pain better: 5	off: U = 154.00,	
	No change: 13	No change: 9	No change: 3	z = 0.657,	
	Pain worse: 5	Pain worse: 5	Pain worse: 1	p = 0.544	
**CPM response**				on: t(53) = 1.326,	
	0.15 +/- 6.01	2.38 +/- 6.40	0.72 +/- 6.72	p = 0.190	t(24) = 1.075,
	[-13.3–13.3]	[-10.6–14.4]	[-18.0–13.9]	off: t(53) = 0.332,	p = 0.293
				p = 0.741	

Results of group comparisons (healthy subjects vs. Parkinson patients(PD) with (on) and without medication (off) and inner group comparisons (on vs. off). The symbol “†” characterizes significant p-values that did not survive Bonferroni correction for multiple testing. UPDRS = Unified Parkinson's Disease Rating Scale; HADS = Hospital Anxiety and Depression Score;

* = data of one patient is missing.

### Experimental parameters and expectation ratings

Mean stimulation temperatures, pain intensity ratings of TS in block 1, cold pain and expectation ratings were comparable between PD patients and controls and also between patients “on” and “off” medication. Both groups expected the pain intensity not to change considerably during the cold pressor task (Table 2).

### CPM responses

CPM responses did not differ between controls and PD patients or between patients “on” and “off” medication (Table 2).

Analyses of group-specific CPM responses using one sample t-tests revealed no significant CPM responses in controls (*t(29) = 0*.*139*, *p = 0*.*890*) and PD patients “off” medication (*t(24) = 0*.*538*, *p = 0*.*596*), whereas patients “on” medication exhibited a trend for a significant CPM response (*t(24) = 1*.*856*, *p = 0*.*076*). A frequency analyses about the number of "inhibitors" (= CPM response > 0) and "facilitators" (= CPM response < 0) revealed that in the “on” condition 15 patients could be classified as “inhibitors” and 10 as “facilitators” (40% facilitators). In the off condition, 13 patients were “inhibitors” and 12 “facilitators” (48% “facilitators”). In the group of healthy controls there were 14 inhibitors and 16 facilitators (53% “facilitators”). The proportion of inhibitors and facilitators was not different between the on and off conditions, nor between patients and healthy controls (*χ² = 0*.*973*, *p = 0*.*324)*.

#### PD subtypes and chronic pain

Kruskal-Wallis tests revealed no significant differences between the three subtypes regarding stimulation temperatures, mean pain intensity ratings in block 1 or “cold pain ratings” in both the “on” and “off” condition (*all p>0*.*1*).

**Fig 2:**
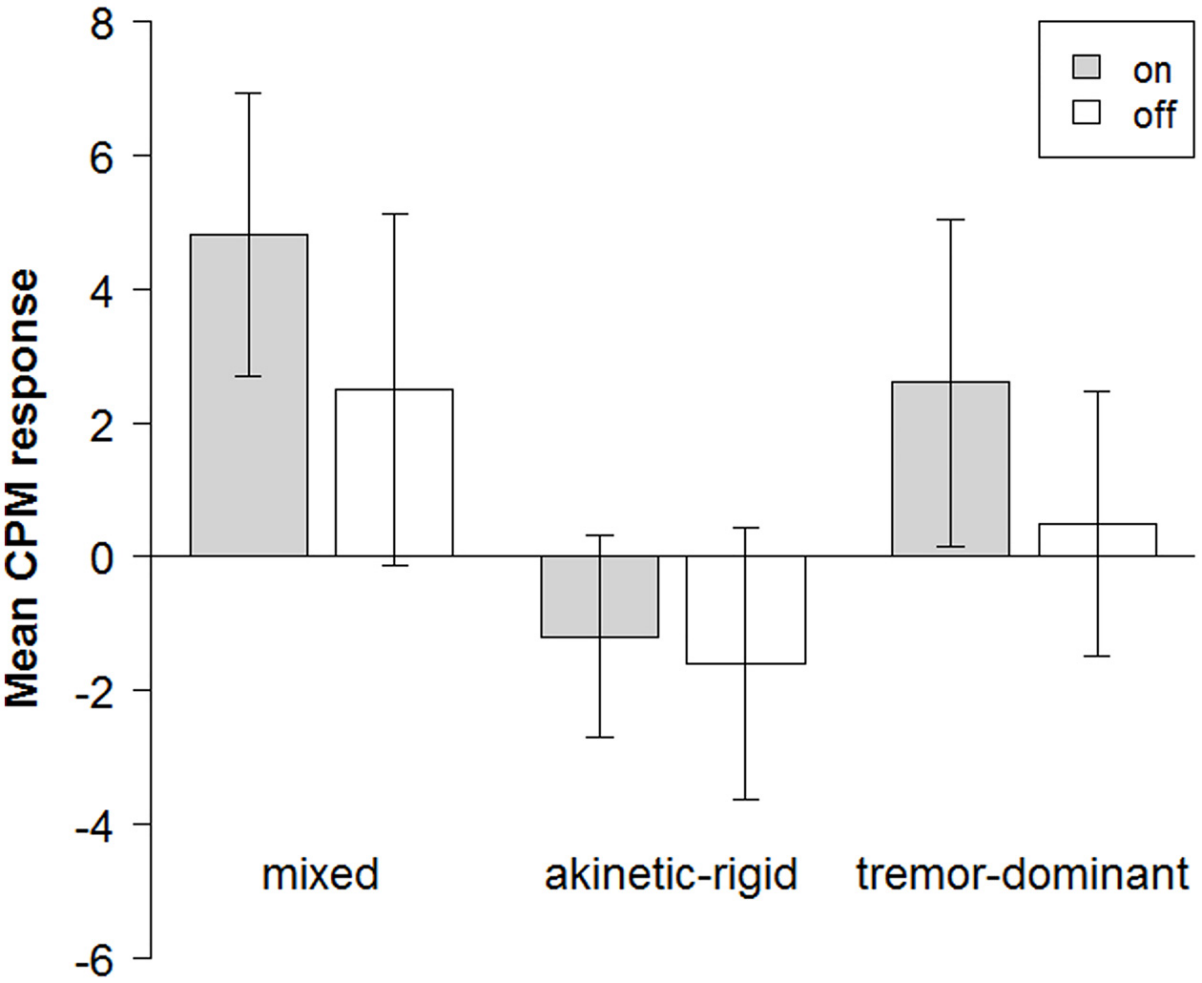
Mean CPM results of PD subtype. Mixed type (left), akinetic-rigid type (middle) and tremor-dominant type (left) in the “on” (light gray) and “off”(dark gray) condition (with standard errors of mean).

Given that there were no significant statistical differences in CPM responses between the “on” and “off” condition we decided to pool data of conditions using the mean CPM response of the “on” and “off” condition of each patient to assess potential subtype differences in CPM magnitude entering one value per subject into the statistical analysis. Kruskal-Wallis tests revealed subtype differences at a trend level (*H(2) = 5*.*596*, *p = 0*.*061*). The tremor-dominant (CPM: “pooled” = 1.5+/-4.6; “on” = 2.6+/-6.9; “off” = 0.5+/-5.6) and mixed type (“pooled” = 3.6+/-6.0; “on” = 4.8+/-6.7; “off” = 2.5+/-8.3) showed positive CPM responses whereas akinetic-rigid patients showed negative CPM results (“pooled” = -1.4+/-4.0; “on” = -1.2+/-4.0; “off”:-1.6+/-5.4; Fig 2).

18 PD patients suffered from pain (5 tremor-dominant, 6 akinetic-rigid, 7 mixed). 10 had chronic pain lasting at least 3 months (4 tremor-dominant, 4 akinetic-rigid, 2 mixed) and 8 reported remittent, but no acute pain (1 tremor-dominant, 2 akinetic-rigid, 5 mixed).

Data of the remaining 7 patients without and 10 patients with chronic pain were compared using Mann-Whitney U tests. Stimulation temperatures, pain intensity ratings in block 1, ice ratings and CPM magnitudes did not differ between both groups in either of the two conditions (“on” and “off”; *all p>0*.*5*). As revealed by one sample t-tests both, patients with chronic pain and without pain, showed no significant CPM responses “on” or “off” medication at all (*all p>0*.*1*). Comparing all patients who reported pain (chronic and remittent n = 18) with those without pain (n = 7) using Mann-Whitney U tests regarding stimulation temperatures, pain intensity ratings in block 1, ice ratings and CPM magnitudes, we could not detect any differences between both groups in any of the two conditions, either (“on” and “off”; *all p>0*.*5*). As revealed by one sample t-tests both, patients with pain (chronic and remittent) and patients without pain showed no significant CPM responses “on” or “off” medication (*all p>0*.*1*).

### Correlations of clinical parameters and CPM

There were no significant correlations between CPM responses and clinical or neuropsychiatric tests in controls or PD patients (Table 3).

**Table 3:** Correlations for Parkinson patients and healthy subjects.

Parameter	Conditioned pain modulation	Conditioned pain modulation
	On (under medication)	Off (without medication)
**PARKINSON PATIENTS**		
** Age**	Pearson´s r = -0.303,	Pearson´s r = 0.000,
	p = 0.141	p = 0.999
** Age at symptom onset**	Pearson´s r = 0.049,	Pearson´s r = 0.360,
	p = 0.828	p = 0.099
** UPDRS Total On** *	Pearson´s r = -0.168,	-
	p = 0.423	
** UPDRS Total Off** *	-	Pearson´s r = -0.124,
		p = 0.556
** UPDRS Motor On** *	Pearson´s r = -0.152,	-
	p = 0.469	
** UPDRS Motor Off** *	-	Pearson´s r = -0.119,
		p = 0.570
** HADS subscale anxiety**	Spearman = -0.255,	Spearman = -0.098,
	p = 0.218	p = 0.642
** HADS subscale depression**	Spearman = -0.018,	Spearman = 0.011,
	p = 0.934	p = 0.957
** Expectation**	Pearson´s r = -0.155,	Pearson´s r = -0.429,
	p = 0.460	p = 0.032†
**HEALTHY SUBJECTS**		
** Age**	Pearson´s r = 0.101,	-
	p = 0.596	
** HADS subscale anxiety**	Spearman = 0.052,	-
	p = 0.897	
** HADS subscale depression**	Spearman = -0.254,	-
	p = 0.176	
** Expectation**	Pearson´s r = 0.033,	-
	p = 0.863	

Results of correlations of study results and clinical and neuropsychiatric parameters for PD patients (upper part) and healthy subjects (lower part). Boxes marked with “-” signify calculations that are not applicable. The symbol “†” characterizes significant p-values that did not survive Bonferroni correction for multiple testing. UPDRS = Unified Parkinson's Disease Rating Scale; HADS = Hospital Anxiety and Depression Score;

* = data of one patient is missing.

## Discussion

In this study we investigated (i) whether CPM responses differ between PD patients and age and gender-matched controls, (ii) whether they are influenced by dopaminergic medication in PD and (iii) whether other PD-specific factors affect CPM responses.

Several important findings derived from this study:

1. No difference in CPM responses was observed between patients and controls.
2. There was no significant effect of dopaminergic medication (“on” vs.”off”) on CPM responses in PD.
3. PD subtype analysis suggests potential CPM differences, with strongest impairment of descending pain inhibition in akinetic-rigid patients.

To our knowledge, only two studies have examined **CPM responses** of PD patients so far [19, 20] and only one tested the influence of dopaminergic medication on CPM [19]. Both studies did not find significant differences in CPM responses compared to controls.

Both patients and controls showed relatively small CPM responses which is consistent with the known age-dependent decline in CPM response [27, 38, 43, 44]. For the paradigm used in this study we could show in a previous study strong CPM responses in young healthy adults but no significant CPM responses in middle-aged and older adults [27]. The assessment of CPM responses might still be a valuable tool to assess pain modulatory activity in this age group as the cold pressure provocation can also increase (instead of decrease) the sensitivity to the test stimuli as a sign of an increased tendency for sensitization, as for instance shown in [42]. To differentiate such a physiological age-dependent decline from a malfunctioning descending pain inhibition in PD we compared CPM responses of PD patients and healthy subjects. Consistent with previous studies [19, 20], we found no significant differences, indicating no substantial additional impairment of descending inhibition in PD. Furthermore, there is evidence that the CPM effect also depends on the paradigm used as mentioned for example by Yarnitsky and colleagues [45]. CPM effects observed in paradigms using the cold pressor task as conditioning stimulus result in different CPM effects than other paradigms using e.g. tonic heat or electrical stimulation as conditioning stimulus [46]. In one of the few studies investigating CPM effects in PD, Mylius and colleagues used a paradigm combining tonic heat and electrical stimulation. In contrast to our results, they could show significant CPM effects in both the PD group (mean age 63.4) and the control group (mean age 67.1) but no significant differences of CPM effects between the both groups. One possible explanation for this difference CPM effects in their study and our study could presumably be the use of different CPM paradigms. In our study we decided to apply a well-established CPM paradigm combinig tonic heat stimuli as test stimuli with the cold pressor task, as the cold pressor task is one of the most commonly used methods as conditioning stimulus in CPM paradigms. Interestingly, although anti-nociceptive properties of dopamine [22, 41, 47] and dopamine agonists [48] have been reported, no significant dopaminergic effect on CPM responses could be found in our PD sample which is congruent with a recent study [19]. Yet, the tendency for a CPM effect in PD during on condition might point towards an antinociceptive effect of the dopaminergic treatment. The literature on the influence of dopaminergic treatment on CPM responses is sparse. To the best of our knowledge only one study has investigated the influence of dopaminergic treatment of PD patients on CPM responses revealing no differences between patients on and off medication [19]. In contrast, the application of apomorphine, a non-specific dopamine agonist, increased CPM responses in healthy volunteers [48].

The effect of dopamine on other experimental pain parameters is rather heterogenous with a tendency towards an analgesic effect of dopamine on pain thresholds [21, 49, 50]. In a study by Treister and colleague no associations were found between dopamine-related genes and endogenous pain modulation measured by both painful and non-painful conditioned pain modulation, whereas serotonin transporter gene polymorphism (5-HTTLPR) was related to pain modulation induced by non-painful conditioned pain modulation [51].

The tendency for a CPM effect during the ‘on’ condition in our study may support the notion that dopamine might have some analgesic effects on experimental pain. However, further studies are needed to specify the influence of dopamine on experimental and clinical pain in larger sample sizes.

Because treatments varied considerably between patients (e.g. levodopa, agonists or drug combinations) it was not feasible to analyze the influence of a dopaminergic treatment alone or to compare the effect of different types of PD drugs on CPM responses. A recent study suggested concentration-depending effects of dopamine: Low concentrations induced anti-nociceptive (D2 receptors) and higher levels pro-nociceptive effects (D1 receptors) [26]. It would thus be interesting to examine whether pain scales with the dopamine level or whether drug-naïve de-novo PD patients at an earlier disease stage exhibiting less degeneration in dopaminergic pathways show different CPM responses compared to long-term PD patients.

The lack of CPM differences in PD could also be explained by an insufficient withdrawal from dopaminergic medication. We used a withdrawal period of at least 12 hours, similarly to Mylius et al. [20] who obtained comparable results. Although the plasma half-life period of dopamine agonists is short (usually several hours) [52] and that of levodopa is generally estimated as 0.7 to 1.4 hours [53], it can last up to 7.9 days [54]. The residual dopaminergic concentration could therefore have induced anti-nociceptive effects and normalized the hypothesized pathologically reduced CPM response in PD patients. Future studies should therefore investigate CPM responses in drug-naïve de-novo patients.

Besides dopamine, the impact of other neurotransmitters (e.g. serotonin and norepinephrine) on descending pain inhibition has been investigated [26, 51]. In a recent study CPM responses were found to predict duloxetine (serotonin-norepinephrine reuptake inhibitor) efficacy in patients with painful diabetic neuropathy which highlights the role of serotonin in descending pain inhibition [55]. It is well-known that neurodegeneration in PD also strongly involves neurotransmitters such as serotonin [56], noradrenalin and glutamate [57] which also affect pain perception. Thus, mechanisms other than dopaminergic transmission might explain the high pain prevalence in PD.

Eventually, the anti-nociceptive effect of dopamine especially on descending inhibition could be very minimal and instead dopamine might principally operate via other—perhaps more cognitive—pain modulating mechanisms such as reward, salience or motivation [58, 59] which were not targeted here.

To our knowledge, this is the first study investigating CPM responses in different **PD subtypes**. Although no statistical difference was observed between PD patients (pooled across subtypes) and controls, we found differences of CPM response differences between the three subtypes at a trend level. Our data suggests akinetic-rigid patients may differ from other subtypes. While tremor-dominant and mixed type patients showed slight CPM responses indicating a pain inhibitory “reserve”, akinetic-rigid patients exhibited negative CPM responses pointing towards pain sensitization during the cold pressor task. This finding suggests potential pain processing differences in different PD subtypes and importantly, might explain the lack of statistical effects when pooling across subtypes. To our knowledge, there is so far no study comparing pain perception and processing between PD subtypes. As musculoskeletal pain, the most common painful sensation in PD [60] with prevalences up to 70% [1, 61], is frequently associated with rigidity [18, 60], akinetic-rigid patients might be more likely to suffer from chronic pain than other subtypes. Given that these patients are also more impaired due to faster disease progression, higher frequency of motor fluctuations and a greater risk of cognitive dysfunction [62] and depression [63] compared to other PD subtypes, it appears conceivable that the more extensive neurodegeneration in this subtype [64] also involves brain areas relevant for pain processing and modulation. Unfortunately, there is no gold standard for the subtype classification [65]. Future studies should include larger patient samples and define PD subtypes optimally using an internationally accepted standardized method (e.g. differentiating between tremor- and non-tremor-dominant subtypes [66] or using UPDRS-based scores as implemented recently [67–69]) that does not exist so far [65].

**Other factors** potentially influencing CPM (e.g. expectation [in accordance with previous findings [27]] or chronic pain) did not show significant effects on CPM response.

There are some limitations regarding the results of our study: The sample size analyzing differences in CPM responses between PD subtypes is rather small. Although the study was not powered to detect significant subgroup differences, we feel that this first evidence for potential differences in CPM responses between PD subtypes helps to draw attention to this interesting observation and to motivate investigations in larger samples in the future. As in a previous study with PD patients studying the endogenous pain modulation [21], we decided to use the same side (right) for the test stimulus as the literature on differences in pain processing in PD depending on the more affected body side is very sparse [19]. To our knowledge only one study by Granovsky et al. [19] has provided first evidence of asymmetric pain processing in PD according to the more affected side: PD patients with predominantly left-sided PD showed an increased sensory response causing hyperalgesia (e.g. an increase of pain ratings for noxious heat 49°C from VAS 70.6 to 77.6) after dopaminergic medication which was not seen in predominantly right-sided PD patients. Regarding CPM effects, no influence of the more affected side could be observed. In another study by Mylius et al. no significant differences of CPM effects between PD patients and healthy controls were found [20] applying the CS always on the less affected side.

## Conclusion

Taken together, our study speaks against the assumption that PD is associated with general deficits in endogenous pain inhibition beyond the known age-related decline. However, we observed descriptive subtype differences in pain inhibitory capacities of PD patients which may explain the absence of statistical differences between controls and patients in our and previous studies. Further studies are needed to explore the role of dopamine in pain modulation in general and the underlying mechanisms of the high prevalence of pain in Parkinson´s disease in particular.

## References

[1] Beiske AG, Loge JH, Ronningen A, Svensson E. Pain in Parkinson's disease: Prevalence and characteristics. Pain. 2009;141(1–2):173–7. 19100686 10.1016/j.pain.2008.12.004

[2] Riley D, Lang AE, Blair RD, Birnbaum A, Reid B. Frozen shoulder and other shoulder disturbances in Parkinson's disease. J Neurol Neurosurg Psychiatry. 1989;52(1):63–6. 2709037 10.1136/jnnp.52.1.63 PMC1032658

[3] Tolosa E, Compta Y, Gaig C. The premotor phase of Parkinson's disease. Parkinsonism Relat Disord. 2007;13 Suppl:S2–7. 17681839 10.1016/j.parkreldis.2007.06.007

[4] Quittenbaum BH, Grahn B. Quality of life and pain in Parkinson's disease: a controlled cross-sectional study. Parkinsonism Relat Disord. 2004;10(3):129–36. 15036166 10.1016/j.parkreldis.2003.12.001

[5] Garcia-Ruiz PJ, Chaudhuri KR, Martinez-Martin P. Non-motor symptoms of Parkinson's disease A review…from the past. J Neurol Sci. 2014;338(1–2):30–3. 10.1016/j.jns.2014.01.002 24433931

[6] Millan MJ. Descending control of pain. Prog Neurobiol. 2002;66(6):355–474. 12034378 10.1016/s0301-0082(02)00009-6

[7] Tracey I, Dickenson A. SnapShot: Pain perception. Cell. 2012;148(6):1308–e2. 10.1016/j.cell.2012.03.004 22424237

[8] Ossipov MH, Dussor GO, Porreca F. Central modulation of pain. The Journal of clinical investigation. 2010;120(11):3779–87. 10.1172/JCI43766 21041960 PMC2964993

[9] Yarnitsky D. Conditioned pain modulation (the diffuse noxious inhibitory control-like effect): its relevance for acute and chronic pain states. Curr Opin Anaesthesiol. 2010;23(5):611–5. 20543676 10.1097/ACO.0b013e32833c348b

[10] Le Bars D, Dickenson AH, Besson JM. Diffuse noxious inhibitory controls (DNIC). I. Effects on dorsal horn convergent neurones in the rat. Pain. 1979;6(3):283–304. 460935 10.1016/0304-3959(79)90049-6

[11] Schouenborg J, Dickenson A. Effects of a distant noxious stimulation on A and C fibre-evoked flexion reflexes and neuronal activity in the dorsal horn of the rat. Brain Res. 1985;328(1):23–32. 3971178 10.1016/0006-8993(85)91318-6

[12] Sprenger C, Bingel U, Buchel C. Treating pain with pain: supraspinal mechanisms of endogenous analgesia elicited by heterotopic noxious conditioning stimulation. Pain. 2011;152(2):428–39. 21196078 10.1016/j.pain.2010.11.018

[13] Moont R, Crispel Y, Lev R, Pud D, Yarnitsky D. Temporal changes in cortical activation during conditioned pain modulation (CPM), a LORETA study. Pain. 2011;152(7):1469–77. 21339052 10.1016/j.pain.2011.01.036

[14] Piche M, Arsenault M, Rainville P. Cerebral and cerebrospinal processes underlying counterirritation analgesia. J Neurosci. 2009;29(45):14236–46. 10.1523/JNEUROSCI.2341-09.2009 19906971 PMC6665061

[15] Borsook D, Upadhyay J, Chudler EH, Becerra L. A key role of the basal ganglia in pain and analgesia—insights gained through human functional imaging. Molecular pain. 2010;6:27 10.1186/1744-8069-6-27 20465845 PMC2883978

[16] Chudler EH, Dong WK. The role of the basal ganglia in nociception and pain. Pain. 1995;60(1):3–38. 7715939 10.1016/0304-3959(94)00172-B

[17] Braak H, Del Tredici K, Rub U, de Vos RA, Jansen Steur EN, Braak E. Staging of brain pathology related to sporadic Parkinson's disease. Neurobiol Aging. 2003;24(2):197–211. 12498954 10.1016/s0197-4580(02)00065-9

[18] Fil A, Cano-de-la-Cuerda R, Munoz-Hellin E, Vela L, Ramiro-Gonzalez M, Fernandez-de-Las-Penas C. Pain in Parkinson disease: a review of the literature. Parkinsonism Relat Disord. 2013;19(3):285–94; discussion 10.1016/j.parkreldis.2012.11.009 23246139

[19] Granovsky Y, Schlesinger I, Fadel S, Erikh I, Sprecher E, Yarnitsky D. Asymmetric pain processing in Parkinson's disease. Eur J Neurol. 2013;20(10):1375–82. 10.1111/ene.12188 23701659

[20] Mylius V, Engau I, Teepker M, Stiasny-Kolster K, Schepelmann K, Oertel WH, et al Pain sensitivity and descending inhibition of pain in Parkinson's disease. J Neurol Neurosurg Psychiatry. 2009;80(1):24–8. 10.1136/jnnp.2008.145995 18653553

[21] Gerdelat-Mas A, Simonetta-Moreau M, Thalamas C, Ory-Magne F, Slaoui T, Rascol O, et al Levodopa raises objective pain threshold in Parkinson's disease: a RIII reflex study. J Neurol Neurosurg Psychiatry. 2007;78(10):1140–2. 17504881 10.1136/jnnp.2007.120212 PMC2117570

[22] Brefel-Courbon C, Payoux P, Thalamas C, Ory F, Quelven I, Chollet F, et al Effect of levodopa on pain threshold in Parkinson's disease: a clinical and positron emission tomography study. Mov Disord. 2005;20(12):1557–63. 16078219 10.1002/mds.20629

[23] Djaldetti R, Shifrin A, Rogowski Z, Sprecher E, Melamed E, Yarnitsky D. Quantitative measurement of pain sensation in patients with Parkinson disease. Neurology. 2004;62(12):2171–5. 15210877 10.1212/01.wnl.0000130455.38550.9d

[24] Tinazzi M, Del Vesco C, Defazio G, Fincati E, Smania N, Moretto G, et al Abnormal processing of the nociceptive input in Parkinson's disease: a study with CO2 laser evoked potentials. Pain. 2008;136(1–2):117–24. 17765400 10.1016/j.pain.2007.06.022

[25] Burkey AR, Carstens E, Jasmin L. Dopamine reuptake inhibition in the rostral agranular insular cortex produces antinociception. J Neurosci. 1999;19(10):4169–79. 10234044 10.1523/JNEUROSCI.19-10-04169.1999 PMC6782709

[26] Benarroch EE. Descending monoaminergic pain modulation: bidirectional control and clinical relevance. Neurology. 2008;71(3):217–21. 10.1212/01.wnl.0000318225.51122.63 18625968

[27] Grashorn W, Sprenger C, Forkmann K, Wrobel N, Bingel U. Age-dependent decline of endogenous pain control: exploring the effect of expectation and depression. PLoS One. 2013;8(9):e75629 10.1371/journal.pone.0075629 24086595 PMC3785470

[28] Hoehn MM, Yahr MD. Parkinsonism: onset, progression and mortality. Neurology. 1967;17(5):427–42. 6067254 10.1212/wnl.17.5.427

[29] Kalbe E, Calabrese P, Kohn N, Hilker R, Riedel O, Wittchen HU, et al Screening for cognitive deficits in Parkinson's disease with the Parkinson neuropsychometric dementia assessment (PANDA) instrument. Parkinsonism Relat Disord. 2008;14(2):93–101. 17707678 10.1016/j.parkreldis.2007.06.008

[30] Zigmond AS, Snaith RP. The hospital anxiety and depression scale. Acta psychiatrica Scandinavica. 1983;67(6):361–70. 6880820 10.1111/j.1600-0447.1983.tb09716.x

[31] Ford B. Parkinson disease: Pain in Parkinson disease: the hidden epidemic. Nat Rev Neurol. 2009;5(5):242–3. 10.1038/nrneurol.2009.50 19488080

[32] Lewis GN, Heales L, Rice DA, Rome K, McNair PJ. Reliability of the conditioned pain modulation paradigm to assess endogenous inhibitory pain pathways. Pain Res Manag. 2012;17(2):98–102. 22518372 10.1155/2012/610561 PMC3393056

[33] Fahn S, Elton R, UPDRS program members. Unified Parkinsons Disease Rating Scale In: Fahn S, Marsden CD, Calne DB, Goldstein M, eds. Recent developments in Parkinsons disease, vol 2 2. Florham Park, NJ: Macmillan Healthcare Information; 1987 p. 153–63.

[34] Arnow BA, Hunkeler EM, Blasey CM, Lee J, Constantino MJ, Fireman B, et al Comorbid depression, chronic pain, and disability in primary care. Psychosom Med. 2006;68(2):262–8. 16554392 10.1097/01.psy.0000204851.15499.fc

[35] Castillo RC, Wegener ST, Heins SE, Haythornthwaite JA, Mackenzie EJ, Bosse MJ. Longitudinal Relationships between Anxiety, Depression, and Pain: Results from a Two Year Cohort Study of Lower Extremity Trauma Patients. Pain. 2013. 10.1016/j.pain.2013.08.025 23994104

[36] Cormier S, Piche M, Rainville P. Expectations modulate heterotopic noxious counter-stimulation analgesia. J Pain. 2013;14(2):114–25. 10.1016/j.jpain.2012.10.006 23260452

[37] Goffaux P, Redmond WJ, Rainville P, Marchand S. Descending analgesia—when the spine echoes what the brain expects. Pain. 2007;130(1–2):137–43. 17215080 10.1016/j.pain.2006.11.011

[38] Lariviere M, Goffaux P, Marchand S, Julien N. Changes in pain perception and descending inhibitory controls start at middle age in healthy adults. Clin J Pain. 2007;23(6):506–10. 17575490 10.1097/AJP.0b013e31806a23e8

[39] Nir RR, Yarnitsky D, Honigman L, Granot M. Cognitive manipulation targeted at decreasing the conditioning pain perception reduces the efficacy of conditioned pain modulation. Pain. 2012;153(1):170–6. 22119318 10.1016/j.pain.2011.10.010

[40] Bjelland I, Dahl AA, Haug TT, Neckelmann D. The validity of the Hospital Anxiety and Depression Scale. An updated literature review. Journal of psychosomatic research. 2002;52(2):69–77. 11832252 10.1016/s0022-3999(01)00296-3

[41] Potvin S, Larouche A, Normand E, de Souza JB, Gaumond I, Grignon S, et al DRD3 Ser9Gly polymorphism is related to thermal pain perception and modulation in chronic widespread pain patients and healthy controls. J Pain. 2009;10(9):969–75. 10.1016/j.jpain.2009.03.013 19464960

[42] Sandrini G, Rossi P, Milanov I, Serrao M, Cecchini AP, Nappi G. Abnormal modulatory influence of diffuse noxious inhibitory controls in migraine and chronic tension-type headache patients. Cephalalgia: an international journal of headache. 2006;26(7):782–9. 16776692 10.1111/j.1468-2982.2006.01130.x

[43] Washington LL, Gibson SJ, Helme RD. Age-related differences in the endogenous analgesic response to repeated cold water immersion in human volunteers. Pain. 2000;89(1):89–96. 11113297 10.1016/S0304-3959(00)00352-3

[44] Edwards RR, Fillingim RB, Ness TJ. Age-related differences in endogenous pain modulation: a comparison of diffuse noxious inhibitory controls in healthy older and younger adults. Pain. 2003;101(1–2):155–65. 12507710 10.1016/s0304-3959(02)00324-x

[45] Yarnitsky D, Arendt-Nielsen L, Bouhassira D, Edwards RR, Fillingim RB, Granot M, et al Recommendations on terminology and practice of psychophysical DNIC testing. Eur J Pain. 2010;14(4):339 10.1016/j.ejpain.2010.02.004 20227310

[46] Pud D, Granovsky Y, Yarnitsky D. The methodology of experimentally induced diffuse noxious inhibitory control (DNIC)-like effect in humans. Pain. 2009;144(1–2):16–9. 19359095 10.1016/j.pain.2009.02.015

[47] Hagelberg N, Jaaskelainen SK, Martikainen IK, Mansikka H, Forssell H, Scheinin H, et al Striatal dopamine D2 receptors in modulation of pain in humans: a review. Eur J Pharmacol. 2004;500(1–3):187–92. 15464032 10.1016/j.ejphar.2004.07.024

[48] Treister R, Pud D, Eisenberg E. The dopamine agonist apomorphine enhances conditioned pain modulation in healthy humans. Neurosci Lett. 2013;548:115–9. 10.1016/j.neulet.2013.05.041 23727387

[49] Conte A, Khan N, Defazio G, Rothwell JC, Berardelli A. Pathophysiology of somatosensory abnormalities in Parkinson disease. Nat Rev Neurol. 2013;9(12):687–97. 10.1038/nrneurol.2013.224 24217516

[50] Lim SY, Farrell MJ, Gibson SJ, Helme RD, Lang AE, Evans AH. Do dyskinesia and pain share common pathophysiological mechanisms in Parkinson's disease? Mov Disord. 2008;23(12):1689–95. 10.1002/mds.22111 18709675

[51] Treister R, Pud D, Ebstein RP, Laiba E, Raz Y, Gershon E, et al Association between polymorphisms in serotonin and dopamine-related genes and endogenous pain modulation. J Pain. 2011;12(8):875–83. 10.1016/j.jpain.2011.02.348 21719351

[52] Brooks DJ. Dopamine agonists: their role in the treatment of Parkinson's disease. J Neurol Neurosurg Psychiatry. 2000;68(6):685–9. 10811688 10.1136/jnnp.68.6.685 PMC1736955

[53] Contin M, Martinelli P. Pharmacokinetics of levodopa. J Neurol. 2010;257(Suppl 2):S253–61. 10.1007/s00415-010-5728-8 21080186

[54] Fahn S, Oakes D, Shoulson I, Kieburtz K, Rudolph A, Lang A, et al Levodopa and the progression of Parkinson's disease. N Engl J Med. 2004;351(24):2498–508. 15590952 10.1056/NEJMoa033447

[55] Yarnitsky D, Granot M, Nahman-Averbuch H, Khamaisi M, Granovsky Y. Conditioned pain modulation predicts duloxetine efficacy in painful diabetic neuropathy. Pain. 2012;153(6):1193–8. 22480803 10.1016/j.pain.2012.02.021

[56] Fox SH, Chuang R, Brotchie JM. Serotonin and Parkinson's disease: On movement, mood, and madness. Mov Disord. 2009;24(9):1255–66. 10.1002/mds.22473 19412960

[57] Brichta L, Greengard P, Flajolet M. Advances in the pharmacological treatment of Parkinson's disease: targeting neurotransmitter systems. Trends Neurosci. 2013;36(9):543–54. 10.1016/j.tins.2013.06.003 23876424

[58] Becker S, Gandhi W, Elfassy NM, Schweinhardt P. The role of dopamine in the perceptual modulation of nociceptive stimuli by monetary wins or losses. Eur J Neurosci. 2013;(38):3080–8. 23841460 10.1111/ejn.12303

[59] Bromberg-Martin ES, Matsumoto M, Hikosaka O. Dopamine in motivational control: rewarding, aversive, and alerting. Neuron. 2010;68(5):815–34. 10.1016/j.neuron.2010.11.022 21144997 PMC3032992

[60] Ford B. Pain in Parkinson's disease. Mov Disord. 2010;25 Suppl 1:S98–103. 10.1002/mds.22716 20187254

[61] Ha AD, Jankovic J. Pain in Parkinson's disease. Mov Disord. 2012;27(4):485–91. 10.1002/mds.23959 21953990

[62] Baumann CR, Held U, Valko PO, Wienecke M, Waldvogel D. Body side and predominant motor features at the onset of Parkinson's disease are linked to motor and nonmotor progression. Mov Disord. 2014;29(2):207–13. 10.1002/mds.25650 24105646

[63] Starkstein SE, Petracca G, Chemerinski E, Teson A, Sabe L, Merello M, et al Depression in classic versus akinetic-rigid Parkinson's disease. Mov Disord. 1998;13(1):29–33. 9452322 10.1002/mds.870130109

[64] Paulus W, Jellinger K. The neuropathologic basis of different clinical subgroups of Parkinson's disease. J Neuropathol Exp Neurol. 1991;50(6):743–55. 1748881 10.1097/00005072-199111000-00006

[65] Marras C, Lang A. Parkinson's disease subtypes: lost in translation? J Neurol Neurosurg Psychiatry. 2013;84(4):409–15. 10.1136/jnnp-2012-303455 22952329

[66] Thenganatt MA, Jankovic J. Parkinson disease subtypes. JAMA neurology. 2014;71(4):499–504. 10.1001/jamaneurol.2013.6233 24514863

[67] Jankovic J, McDermott M, Carter J, Gauthier S, Goetz C, Golbe L, et al Variable expression of Parkinson's disease: a base-line analysis of the DATATOP cohort. The Parkinson Study Group. Neurology. 1990;40(10):1529–34. 2215943 10.1212/wnl.40.10.1529

[68] Eggers C, Pedrosa DJ, Kahraman D, Maier F, Lewis CJ, Fink GR, et al Parkinson subtypes progress differently in clinical course and imaging pattern. PLoS One. 2012;7(10):e46813 10.1371/journal.pone.0046813 23056463 PMC3466171

[69] Kang GA, Bronstein JM, Masterman DL, Redelings M, Crum JA, Ritz B. Clinical characteristics in early Parkinson's disease in a central California population-based study. Mov Disord. 2005;20(9):1133–42. 15954133 10.1002/mds.20513 PMC3643967

